# Implication of Post-Induction Minimal Residual Disease in the Management of Pediatric Acute Lymphoblastic Leukemia in a Resource-Limited Setting

**DOI:** 10.7759/cureus.100545

**Published:** 2026-01-01

**Authors:** Waqar Mushtaq, Saadia Anwar, Safwan Ahmad, Mahwish Faizan, Sana Gull, Aimen Gull

**Affiliations:** 1 Hematology and Oncology, University of Child Health Sciences, Lahore, PAK; 2 Pediatric Hematology and Oncology, Children's Hospital and Institute of Child Health, Lahore, PAK; 3 Pediatric Hematology and Oncology, Children's Hospital and Institute of Child Health, Multan, PAK; 4 Pediatric Oncology, Children's Hospital and Institute of Child Health, Lahore, PAK; 5 Hematology and Oncology, Children's Hospital Lahore, Lahore, PAK

**Keywords:** acute myeloblastic leukemia, evaluation, mrd, outcomes, patients

## Abstract

Background: Minimal residual disease (MRD) measurement in pediatric acute lymphoblastic leukemia (ALL) is the most powerful tool for treatment response assessment.

Objective: This study aims to assess the implications of post-induction Day 29 MRD evaluation in the management of pediatric ALL within a resource-limited setting and its correlation with interim Day 8/15 bone marrow assessment.

Materials and methods: A prospective analytical study was conducted at the pediatric hematology oncology department from January 1​​​, ​2022, to December 31​​​​​, 2022. Flowcytometry-based newly diagnosed ALL patients aged 1-16 years were included. MRD assessment on Day 29 of induction and interim bone marrow evaluations on Day 8/15 were performed according to the UKALL-2019 protocol.

Results: Out of 578 patients, 407 (70.4%) were males, and 171 (29.6%) were females. The predominant age group was 5-10 years, including 324 (56.0%) patients. Flow cytometry revealed pre-B ALL in 468 (81.0%) and pre-T ALL in 110 (19.0%). High-risk patients receiving four-drug induction numbered 379 (65.6%), while 199 (34.4%) were standard risk. Interim bone marrow response was M2 in 502 (86.9%) and M3 in 73 (12.6%). Post-induction Day 29 MRD was < 0.01% (negative) in 495 (85.6%) patients who continued the same regimen, and >0.01% (positive) in 83 (14.4%) requiring treatment escalation. Among those with M3 interim marrow response, 42 (7.3%) were high-risk and MRD-positive. Induction mortality occurred in 32 (5.5%) patients, and treatment abandonment occurred in 46 (8.0%).

Conclusion: Our study has demonstrated significant induction remission response in ALL by achieving negative MRD, which correlates strongly with interim bone marrow assessment, proposing Day 29 MRD as a single best cost-effective tool for response evaluation in resource-limited settings.

## Introduction

Acute lymphoblastic leukemia (ALL) is the most common cancer in childhood, with a prevalence of up to 25% of cancers in children who are under the age of 16 years [1]. The survival of ALL has improved significantly over the decades, reaching up to about 90% in the developing world, and it is attributed to an advanced diagnostic approach, finer risk stratification criteria with risk-adapted treatment, better supportive care, and new treatment modalities [2,3]. They are initially stratified based on age, total leucocyte count (TLC), and immunophenotype into standard and high-risk treatment groups, and induction treatment is started, including dexamethasone, vincristine, asparaginase, and intrathecal (IT) methotrexate (three drugs) in standard-risk patients, with the addition of anthracycline drugs in high-risk ALL patients [4]. Risk stratification of ALL patients is based on age, initial TLC, and immunophenotype [3].

In addition to cytogenetic analysis, response to treatment is one of the most important predictors of outcome and a determinant of further treatment group allocation in ALL. Treatment response is assessed on Day 8/15 as per the treatment regimen, and remission is assessed on Day 29 of induction by bone marrow biopsy and MRD [5]. Morphologic CR (mCR) is defined as less than 5% blasts in the regenerated bone marrow. Even after using a risk-adapted therapy and achieving mCR, relapse can still occur. The source of these relapses is the persistence of minimal residual disease (MRD), which is undetectable by standard diagnostic techniques [6]. MRD is considered to be an important emerging marker for disease relapse and treatment monitoring in patients with acute lymphoblastic leukemia [3]. It is defined as the presence of sub-microscopic levels of leukemic cells [7]. There are two methods to detect MRD, either by polymerase chain reaction or by flow cytometry [8]. There is a paucity of data from Pakistan on MRD evaluation and its correlation with interim bone marrow assessment in ALL. Therefore, the present study is undertaken with the objective of determining the significance of MRD for remission assessment and further allocation of the treatment regimen. There is limited local evidence on how interim bone marrow response correlates with MRD in pediatric ALL, and whether Day-29 MRD can reliably guide treatment decisions in resource-limited settings. Most centers still depend on morphology alone, despite its inability to detect sub-microscopic disease.

Objective

The study aimed to analyze the correlation between interim bone marrow response (Day 8/15) and MRD status in pediatric ALL and to evaluate the significance of post-induction Day 29 MRD assessment in guiding further treatment allocation.

## Materials and methods

This was a prospective analytical study conducted in the pediatric oncology department, University of Child Health Sciences/The Children’s Hospital, Lahore, Pakistan, from January 1, 2022, to December 31, 2022. A total of 578 newly diagnosed pediatric ALL patients aged 1-16 years were included. All participants were diagnosed through flow cytometry and received induction chemotherapy according to risk stratification. Patients underwent early bone marrow assessment on Day 8 or Day 15, depending on their treatment regimen, and MRD evaluation on Day 29 of induction. Both genders were included. Patients younger than one year or older than 16 years, those diagnosed without flow cytometry, cases of lymphoblastic lymphoma treated under the UKALL protocol, previously treated patients, and those without interim or Day-29 MRD assessment were excluded from the study.

Data collection

The Institutional Review Board (IRB) of the University of Child Health Sciences/Children’s Hospital Lahore, Pakistan, approved the study. After obtaining informed consent, patients were enrolled according to the inclusion criteria through the emergency (ER) and outpatient departments (OPD). Age, gender, presenting complaints, bone marrow characteristics after 8/15 days of induction according to treatment regimen, and the presence or absence of MRD after 29 days of induction were recorded on a comprehensive proforma. The patients were then shifted to the ward where the initial workup in the form of baseline and tumor lysis syndrome was performed, and the patients were booked for flow cytometry either on peripheral smear or bone marrow, depending on their risk stratification as per National Cancer Institute (NCI) criteria. Subsequently, after receiving the flow cytometry report, the chemo protocol was started as either regimen A or regimen B as per their risk stratification (NCI criteria).

After the initial treatment of induction therapy, the patients were discharged to the outpatient chemo department, where they were observed for the side effects of chemotherapy. They were followed in both the outpatient department and the chemotherapy bay. Their interim assessment bone marrow biopsy was performed on Day 8 in regimen B and Day 15 in regimen A. However, their post-induction bone marrow biopsy, along with MRD, was done on Day 29 of the induction therapy. The MRD facility was not available inside the hospital and was outsourced. MRD testing was performed via eight-color flow cytometry at an accredited external reference laboratory. The cost of MRD was 10,000 PKR. The next regimen was assigned after the review of MRD. If the MRD was in remission, the same regimen was used; if not in remission, the treatment was escalated to the next regimen.

Statistical analysis

Results were assessed by using SPSS version 27 (IBM Corp., Armonk, NY). Quantitative variables will be expressed as means and standard deviations (SD). Frequencies will be given for qualitative variables. Chi-square test will be applied on age and gender. Quantitative variables were expressed as mean ± SD, while qualitative variables were presented as frequencies and percentages. Chi-square tests were applied for categorical comparisons. Logistic regression modeling was performed to identify independent predictors of MRD positivity, adjusting for age, gender, baseline TLC, immunophenotype, and early marrow response. A p-value < 0.05 was considered statistically significant.

## Results

A total of 578 patients were enrolled in the study. There was a clear male predominance, with 407 (70.4%) males and 171 (29.6%) females, giving a male-to-female ratio of 2.4:1. The overall mean age was 8.16 years, ranging from 2 to 16 years. The most prevalent age group was 5-10 years, comprising 285 (49.3%) patients. Among males, 171 (42%) were classified as high-risk, while 76 (44.4%) females were categorized as high-risk. In the 5-10-year age group, 91 (31.9%) patients fell into the high-risk category, highlighting that younger age did not necessarily predict a lower-risk disease profile (Table 1).

**Table 1:** Demographic and baseline characteristics (n = 578) Data are presented as numbers (percentages) and mean ± standard deviation.

Characteristics	Values
Total patients	578
Male	407 (70.4%)
Female	171 (29.6%)
Male:female ratio	2.4:1
Mean age (years)	8.16
Age range (years)	2–16
Most prevalent age group (5–10 years)	285 (49.3%)
Risk stratification	
Male high-risk	171 (42.0%)
Female high-risk	76 (44.4%)
5–10 years high-risk	91 (31.9%)

Most patients, 231 (39.9%), presented with TLC < 10,000/µL, of which 41 (17.7%) were classified as high-risk. A total of 165 patients had TLC between 10,000 and 50,000/µL, and 64 (38.7%) of these were high-risk. Notably, all 182 (31.4%) patients with TLC > 50,000/µL were categorized as high-risk, showing a strong correlation between high TLC and high-risk classification. Flow cytometry revealed that pre-B ALL was the most common subtype, seen in 487 (84.3%) patients, out of which 216 (44.3%) were high-risk. Pre-T ALL was present in 91 (15.7%) patients. This distribution reinforced the predominance of pre-B ALL in the pediatric cohort (Table 2).

**Table 2:** Risk stratification by total leukocyte count (TLC) Data are shown as numbers (percentages). Association between TLC category and risk status was tested using the Chi-square test (χ² = 289.5, p < 0.001). p < 0.05 is considered statistically significant.

TLC count (cells/µL)	Total patients (n)	High-risk (n, %)
<10,000	231	41 (17.7%)
10,000–50,000	165	64 (38.7%)
>50,000	182	182 (100%)
Immunophenotype		
Pre-B ALL	487 (84.3%)	216 (44.3%)
Pre-T ALL	91 (15.7%)	—

The interim bone marrow response was predominantly M2 in 502 (86.9%) patients, with 185 (36%) in the standard-risk group and 317 (63%) in the high-risk group. M3 marrow was found in 73 (12.6%) patients, mostly high-risk (83.6%). Only three (0.1%) patients showed M1 marrow at interim evaluation. Post-induction marrow evaluation showed M1 in 438 (75.8%), M2 in 58 (10%), and M3 in 82 (14.2%) cases. Day-29 MRD assessment revealed <0.01% positivity in 363 (62.8%) patients, who continued on their initial regimen. Conversely, 30 (8.4%) patients had MRD > 0.01% and required treatment escalation. MRD positivity was strongly associated with interim M3 marrow and post-induction positive BM (24.2%). The overall remission rate was 91.6% (Table 3).

**Table 3:** Bone marrow and MRD response Values are expressed as n (%). The association between marrow status and MRD level was analyzed using the chi-square test (χ² = 31.64, p < 0.001). A p-value < 0.05 was considered statistically significant.

Response type	Category	Total (n, %)	Standard risk (n, %)	High risk (n, %)
Interim BM	M1	3 (0.1%)	2	1
	M2	502 (86.9%)	185 (36%)	317 (63%)
	M3	73 (12.6%)	12 (16.4%)	61 (83.6%)
Post-induction BM	M1	438 (75.8%)	—	—
	M2	58 (10.0%)	—	—
	M3	82 (14.2%)	—	—
Day-29 MRD	<0.01% (Negative)	363 (62.8%)	—	—
	>0.01% (Positive)	30 (8.4%)	—	—

In the standard-risk group (regimen A, n = 271), remission was achieved in 249 patients (91.9%), while five (1.8%) required treatment escalation. Induction mortality occurred in 12 patients (4.4%), and abandonment was reported in five cases (1.9%). In the high-risk group (regimen B, n = 307), remission was observed in 278 patients (90.6%), with 25 patients (8.1%) requiring escalation of therapy. Induction mortality was considerably higher at 73 patients (23.8%), and 13 patients (4.2%) abandoned treatment. When analyzed according to MRD status, patients with MRD less than 0.01% (n = 363) had remission in 360 cases (99.2%), with only two deaths (0.6%) and one case of abandonment (0.2%), and none required therapy escalation. In contrast, patients with MRD greater than 0.01% (n = 30) had remission in 18 cases (60.0%), while 12 (40.0%) required escalation of therapy. Mortality was higher in this group at five cases (16.7%), and abandonment occurred in two patients (6.7%). The overall remission rate was 91.6% (Table 4).

**Table 4:** Treatment outcomes by risk and MRD status The association between MRD and treatment outcome was analyzed using the Chi-square test (χ² = 45.82, p < 0.001). Statistical significance was considered at p < 0.05.

Category	Total (n)	Remission, n (%)	Escalated therapy, n (%)	Induction mortality, n (%)	Abandonment, n (%)
Standard risk (regimen A)	271	249 (91.9%)	5 (1.8%)	12 (4.4%)	5 (1.9%)
High risk (regimen B)	307	278 (90.6%)	25 (8.1%)	73 (23.8%)	13 (4.2%)
MRD < 0.01%	363	360 (99.2%)	—	2 (0.6%)	1 (0.2%)
MRD > 0.01%	30	18 (60.0%)	12 (40.0%)	5 (16.7%)	2 (6.7%)

## Discussion

This study was conducted to examine the implications of MRD in a resource-limited public hospital in Pakistan. In the literature search, a paucity of recent local data was found on this subject. Our study used similar cutoffs of MRD and also confirmed that MRD can be used as a measure of disease burden, as observed in other international studies. In contrast to other published studies, the mean age of presentation (5-10 years) in our study was 8.16 years, which was different from most of the others, and most of them were stratified in the high-risk group. However, the preponderance of male population, type of leukemia (mainly pre-B-ALL), and the results of post-induction MRD and M1 status on D29 BMB in our study were similar to other published data [9]. The mortality rate was also considerably higher compared to a study from the neighboring country, which showed an induction mortality of 6.57% [7]. This higher incidence could be attributed to the fact that 34.4% of the patients were in the high-risk group, and it remains a significant challenge for patients in LMIC. TLC count was less than 50,000 in 78% of patients compared to 59.2% in a recent local study [10].

The frequency of BCR-ABL translocation was 1.7% in our study, similar to that shown by Gökbuget ​​​​​​et al. (1.3%) [2], but was significantly lower in our study as compared to a recent local study with an incidence as high as 10% [8]. A Romanian study demonstrated an incidence of 2.7% of BCR-ABL1 translocation in their pediatric population [11]. The interim bone marrow at Day 15 showed remission for 86% patients, which was very similar to 83% shown by Qamar ​​​​​​et al. [12]. In a resource-limited country, it is essential to risk-stratify patients early in the treatment of ALL. Our study supported the fact that MRD is a very important tool in predicting prognosis after induction therapy in a resource-limited setting. Although our study did not evaluate the relationship between post-induction MRD positivity and risk factors, a local study did not find a significant association [11]. In our study, 14.4% children required treatment escalation after post-induction MRD; however, currently there are no reliable data on the effect of post-induction MRD status on survival [13]. We also emphasize the fact that with the availability of genetic analysis, further local studies should be carried out to evaluate genomic analyses and MRD together for risk-directed treatment of childhood ALL as a step forward.

Limitations

The study was conducted in a single tertiary care public hospital, which may limit the generalizability of the findings to other healthcare settings. Genetic and cytogenetic profiling could not be performed in all patients due to resource constraints, potentially affecting the depth of risk stratification. Long-term survival and event-free survival data were not evaluated, preventing outcome correlation beyond induction. Furthermore, the study did not assess socioeconomic or treatment adherence factors, which could influence mortality and abandonment rates. Future multicenter studies integrating genomic profiling with MRD analysis are recommended to better define risk-adapted treatment protocols in pediatric ALL within resource-limited environments.

## Conclusions

It is concluded that risk stratification and MRD status at Day 29 are strong predictors of treatment outcomes in pediatric ALL. Patients in the standard-risk group and those with MRD negativity achieved higher remission rates with minimal mortality, while high-risk and MRD-positive patients experienced poorer outcomes, greater need for treatment escalation, and higher induction mortality. These findings emphasize the importance of incorporating MRD assessment into routine clinical practice and highlight the need for tailored treatment strategies and enhanced supportive care, especially in high-risk patients managed in resource-limited settings.
